# Investigating the relationship between microbial network features of giant kelp “seedbank” cultures and subsequent farm performance

**DOI:** 10.1371/journal.pone.0295740

**Published:** 2024-03-27

**Authors:** Melisa G. Osborne, Ariel Levi Simons, Gary Molano, Bernadeth Tolentino, Anupam Singh, Gabriel J. Montecinos Arismendi, Filipe Alberto, Sergey V. Nuzhdin

**Affiliations:** 1 Department of Molecular and Computational Biology, University of Southern California, Los Angeles, CA, United States of America; 2 Department of Ecology and Evolutionary Biology, University of California Santa Cruz, Santa Cruz, CA, United States of America; 3 Department of Marine and Environmental Biology, University of Southern California, Los Angeles, CA, United States of America; 4 Department of Biological Sciences, University of Wisconsin-Milwaukee, Milwaukee, WI, United States of America; University of Salento Department of Biological and Environmental Sciences and Technologies: Universita del Salento Dipartimento di Scienze e Tecnologie Biologiche ed Ambientali, ITALY

## Abstract

Microbial inoculants can increase the yield of cultivated crops and are successful in independent trials; however, efficacy drops in large-scale applications due to insufficient consideration of microbial community dynamics. The structure of microbiomes, in addition to the impact of individual taxa, is an important factor to consider when designing growth-promoting inoculants. Here, we investigate the microbial network and community assembly patterns of *Macrocystis pyrifera* gametophyte germplasm cultures (collectively referred to as a “seedbank”) used to cultivate an offshore farm in Santa Barbara, California, and identify network features associated with increased biomass of mature sporophytes. We found that [1] several network features, such as clustering coefficient and edge ratios, significantly vary with biomass outcomes; [2] gametophytes that become low- or high-biomass sporophytes have different hub taxa; and [3] microbial community assembly of gametophyte germplasm cultures is niche-driven. Overall, this study describes microbial community dynamics in *M*. *pyrifera* germplasm cultures and ultimately supports the development of early life stage inoculants that can be used on seaweed cultivars to increase biomass yield.

## Introduction

Microbes have a significant impact on plant physiology, and there has been a wealth of research on the use of microbial inoculants (i.e., the introduction or addition of beneficial bacteria to a host) in agriculture [1–9]. Previous work has shown that addition of growth-promoting bacteria can increase the overall health and production of several agricultural crops including rice, maize, and cotton [1–3]. In particular, use of these inoculants at an early life stage in plant hosts can increase crop yield and farm productivity [4–9]. Many studies focus on the impact that individual microbes have on host health. While useful, this approach is limited because it does not sufficiently consider that host microbiomes (i.e., the collection of microbiota native to a host) are not a collection of isolated microbes, but rather an interdependent group with complex functional and metabolic pathways [10]. The use of microbial inoculants can disturb these pathways, and have unintended effects on plant hosts. Microbial inoculants can compete with native species, preventing successful colonization of the inoculant or causing negative impacts on crop performance [11]. Inoculants may also prompt microbial succession, thereby altering community structure and function [12,13]. Understanding how microbial networks are naturally structured can increase efficacy of microbial inoculants in large-scale agricultural applications [6,14]. Therefore, in order to fully harness the beneficial impact of microbes and establish a strong framework for growth-promoting inoculants, it is critical to understand microbial networks and community dynamics in the context of crop outcomes.

Microbial community dynamics of host-associated microbiomes may be better understood by analyzing co-occurrence networks, hub microbes, and community assembly patterns. Co-occurrence networks represent the likely patterns of spatial co-occurrence (i.e., being present together in an environment), which can be used to infer potential relationships between individual taxa. These networks can be visually represented as a collection of nodes and edges. In the context of this study, nodes represent unique taxa and edges represent the links or co-occurrence patterns between them. Co-occurrence patterns can be quantified with measures of network topology such as the clustering coefficient, modularity, and edge ratios. Clustering coefficient and modularity describe the division of a network into sub-networks and the density of connections between nodes, respectively. The ratio of positive to negative edges, which represent significant patterns of spatial co-occurrence or exclusion, can also indicate the degree to which the community has potentially synergistic or competitive interactions. By investigating how microbial co-occurrence networks at the early life stage of crops varies with crop performance we may use this insight to predict crop yield and develop agricultural inoculants that synergize with network features of high-performing crops [10,12].

Hub microbes are central to the process of microbiome recruitment and have several associations across the microbial network [15–17]. They are defined as having a disproportionate number of links with other taxa in the network. Hub microbes are key drivers of the overall microbial community because of their intrinsic ability to recruit and support the introduction of other bacteria that directly benefit the host, particularly at the early life stage of crops [6]. The impact hub microbes have on the diversity of host microbiomes can occur directly (i.e., by impacting the colonization of other microbes) or indirectly (i.e., through the host) [17]. Hub microbes of high-performing crops can be inoculated in tandem with growth-promoting bacteria to improve crop fitness by increasing native recruitment of beneficial bacteria and supporting synergistic interactions [6]. Furthermore, the use of inoculants that do not compete with hub taxa can also improve long-term success and facilitate predictable changes in the overall community [5].

While co-occurrence network and hub microbe analysis, as described above, can be used to understand representative microbiomes for a group of hosts, community assembly patterns provide insight into what mechanisms drive variation of microbiomes across hosts. The two most common forms of community assembly follow a stochastic or niche assembly process [18]. During stochastic assembly, microbes are randomly incorporated from the environment into a community. During niche assembly, the likelihood of species being incorporated is dependent on their ecological role and those of existing community members. Here, we investigate the relative likelihood of these two assembly processes using the zeta diversity framework, a method for calculating the number of shared species across an arbitrarily large number of sample sites [18,19]. As the number of sites being compared increases, zeta diversity typically decays following an exponential or power-law form [20,21]. An exponential decay suggests that communities are more likely to be assembled stochastically, while a power-law decay suggests they are more likely to be assembled via niche-differentiation [19]. In the context of this study, understanding whether microbial communities assemble in a stochastic or niche-driven manner can help improve inoculant design. If the assembly is niche-driven, for example, inoculants can be designed to avoid competition with established niches and increase likelihood of success.

**Fig 1:**
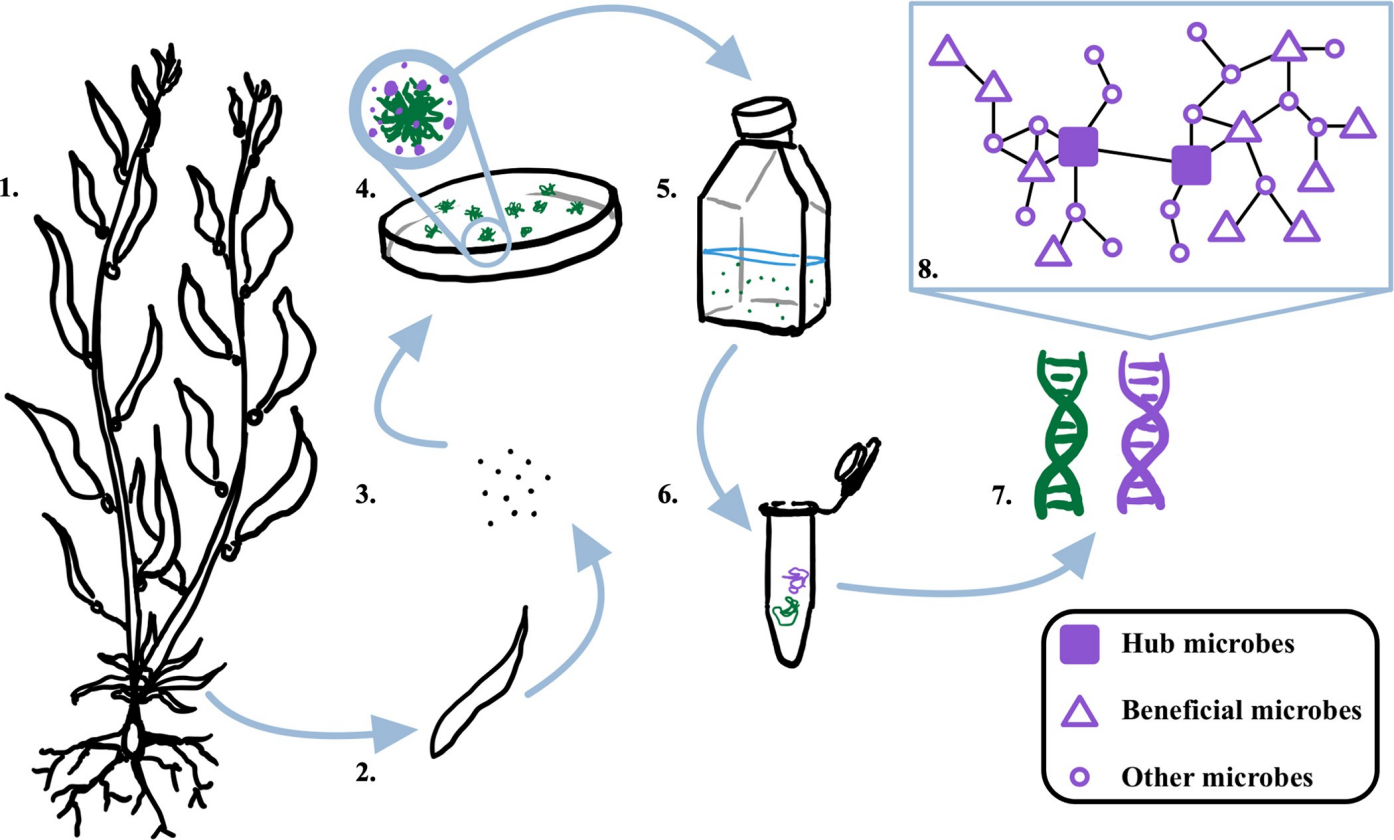
Workflow for data collection and network construction. **1.** Wild *M*. *pyrifera* sporophylls were collected from four natural populations across Southern California: Arroyo Quemado (AQ), Catalina Island (CI), Camp Pendleton (CP), and Leo Carillo (LC). **2.** Reproductive blades were surface sterilized and prepared for spore release. **3.** Spores were released in sterile Provasoli enriched seawater medium (PES). **4.** Spores were raised to gametophyte stage in petri dishes. Single, genetically unique gametophytes (green) were isolated and used to establish a giant kelp seedbank. No antibiotic treatment was applied, and resident microbes (purple) persisted. **5.** Genetically unique gametophyte germplasm cultures were grown vegetatively in sterile PES. **6.** *M*. *pyrifera* (green) and microbial (purple) DNA of each genetically unique gametophyte culture were co-extracted, followed by shotgun sequencing using an Illumina S4 Novaseq platform. **7.** Microbial DNA was filtered and characterized using the ‘metaxa2’ program with the SILVA128 database. **8.** Microbial networks were constructed and analyzed using the ‘SpiecEasi’ and ‘igraph’ programs.

**Fig 2:**
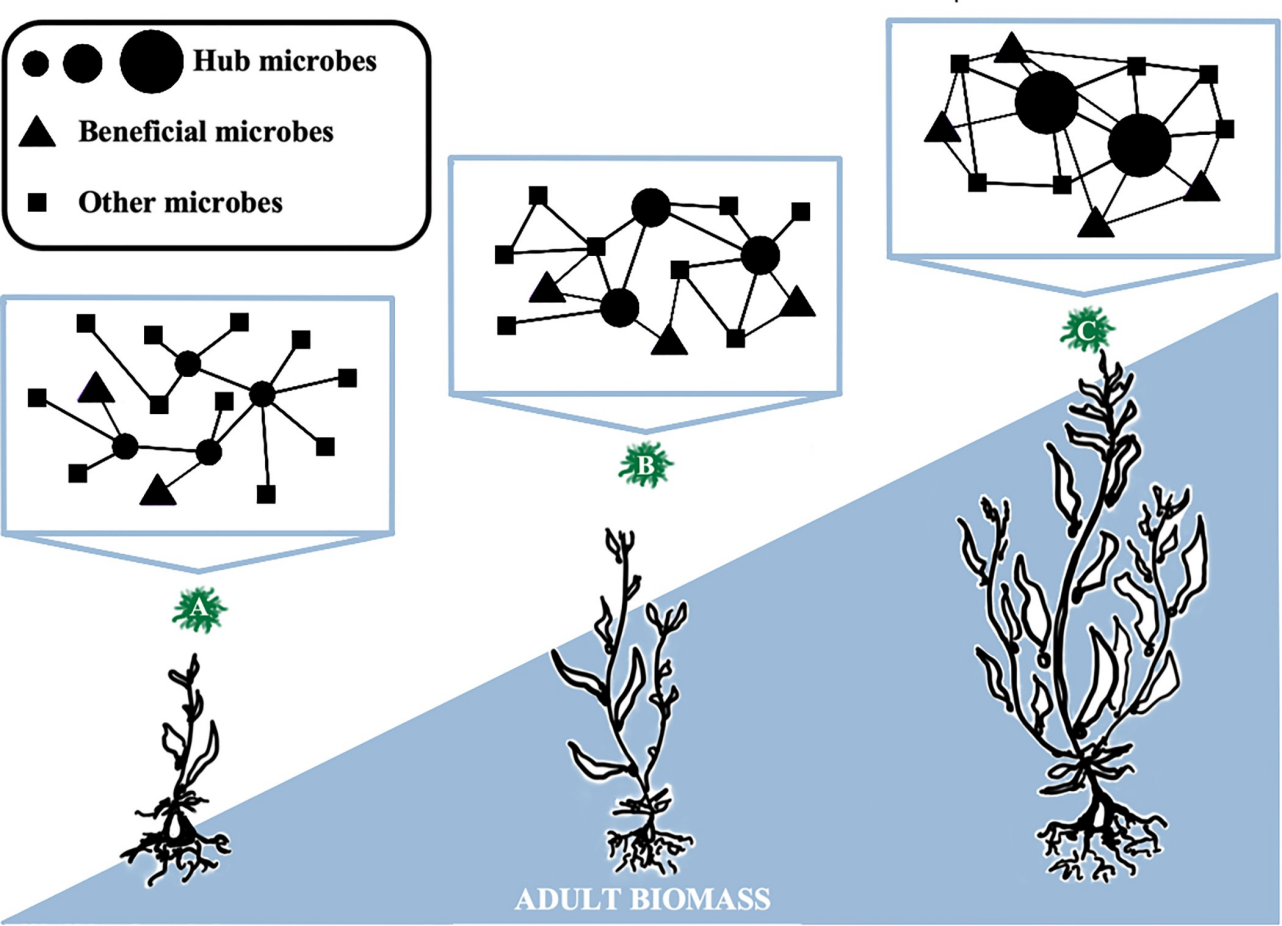
Microbial network features of gametophytes vary with sporophyte biomass. The network features of genetically unique gametophytes (green, labelled A-C) were analyzed to identify whether any characteristics of early stage gametophyte microbiomes are associated with sporophyte biomass.

Analyzing microbial community dynamics, using the methods described above, will allow for more precise development of inoculants that can increase crop yield [12]. Here, we pursue this work with giant kelp (*Macrocystis pyrifera*), a high-potential feedstock for biofuels. This study examines *M*. *pyrifera* at two life stages: a juvenile state named the “gametophyte” stage and a fully mature adult state named the “sporophyte” stage. Our group has previously determined that there is a significant difference in microbial community composition between gametophytes that become high- versus low-biomass sporophytes, and that bacteria within the *Mesorhizobium* genus are key candidates for creating a growth-promoting inoculant [22]. This study builds upon that work by investigating both the topology of microbiome co-occurrence networks, as well as the relative likelihoods of two common community assembly processes for giant kelp seedbank cultures, and the relationships of these network features with the final biomass yield of mature sporophytes. We hypothesize that the final yield of *M*. *pyrifera* adult sporophytes is correlated with differences in microbial community dynamics during the gametophyte stage. We further hypothesize that given the tight ecological interactions between microbes and their seaweed hosts [23], that seedbank microbial communities will assemble through niche-differentiation. Overall, this work provides a valuable knowledge base for developing, and increasing the efficacy of, microbial inoculants used in seaweed aquaculture (Figs 1 and 2).

## Materials and methods

### Ethics statement

This study was carried out with non-destructive sampling in accordance with a scientific collecting permit administered by the State of California Department of Fish and Wildlife (Permit ID: S‐183050002‐18305‐001).

### Production of gametophytes and cultivation of sporophytes

Sporophyte collection, spore release, sequencing, and classification followed protocol reported in Osborne et al. [22] and is briefly described here. Reproductive blades of *M*. *pyrifera* were collected from Southern California regions in December 2018 representing four genetically distinct natural populations [24]: Arroyo Quemado (AQ), Catalina Island (CI), Camp Pendleton (CP), and Leo Carillo (LC). Blades were shipped overnight to University of Wisconsin-Milwaukee for spore release in sterile Provasoli enriched seawater medium (PES) [25] at 34 PSU salinity following the Oppliger method [26]. Spores were raised to the gametophyte stage in a growth chamber under red light (20 μmol photons m^-2^s^-1^) with a 12:12h (Light:Dark) photoperiod at 12°C, then isolated and vegetatively grown under red light (30 μmol photons m^-2^s^-1^) with a 12:12h (Light:Dark) photoperiod at 12°C to create 559 genetically unique germplasm cultures. Increased light intensity at this stage was used to induce faster vegetative growth. From this germplasm, 500 female gametophytes (345 from LC, 54 from AQ, 45 from CI, and 56 from CP) were crossed with a single male from LC (five replicates each) to produce a total of 2,500 gametophyte crosses. These crosses were seeded on polyvinyl lines and grown to the sporophyte stage under white light (60 μmol photons m^-2^s^-1^) with a 16:8h (Light:Dark) photoperiod at 12°C for one month before being shipped overnight to a marine laboratory at the University of California, Santa Barbara (UCSB). Juvenile sporophytes were adjacently planted on ten longlines in an offshore farm 1-mile off the coast of Santa Barbara in May 2019. All surviving sporophytes were harvested between September 7–12, 2019 using Santa Barbara Mariculture’s vessel *Perseverance*. Harvested sporophytes were briefly spin-dried by hand before weighing to record total biomass, which includes stipe and blades. The average biomass of all surviving genetic replicates was calculated and used in this study. A number of individuals were lost due to issues during harvest or premature loss. Due to smaller sample size and restricted availability of phenotype data for the AQ, CI, and CP populations, we only report network analysis across biomass outcomes for the LC population (see: ‘Grouping gametophytes and taxonomy levels for biomass and population comparisons’).

### DNA extraction, microbial shotgun sequence data, and classification

For DNA extraction, aliquots of each gametophyte culture was centrifuged to obtain 50-100mg of gametophyte tissue biomass, which was pulverized using liquid nitrogen. Kelp genome and microbial DNA were co-extracted and sequenced from female and male gametophytes using the NucleoSpin 96 Plant Kit (Macherey-Nagel, Duren, Germany). Gametophyte samples were not treated with an antibiotic prior to DNA extraction; therefore, the microbial DNA of both exogenous and endogenous species was extracted. Sequencing (150bp paired-end) was done at BGI North American NGS lab using an Illumina S4 Novaseq platform and generated approximately 11.2GB or 87 million reads per sample. WGS was chosen over 16S amplicon sequencing to support an umbrella giant kelp breeding project and provide an opportunity to perform functional analysis in future studies. Raw fastq files were processed with the ‘fastp’ program (version 0.20.1) [27]. Due to evidence of bacterial contamination in existing brown macroalgae genomes [28], all reads were included in the bacterial classification pipeline to ensure that all candidate sequences were analyzed. Reads were classified using the ‘metaxa2’ package (version 2.2.2) which extracts and classifies partial rRNA sequences against the SSU_SILVA128 database [29,30]. This version of the database was used to facilitate a comparison of findings between this and a previous study [22]. The resulting abundance table was further processed and analyzed with the ‘phyloseq’ package (version 1.34.0) in R [31,32]. Abundance counts were processed by removing singletons and doubletons, normalizing counts by sequencer, averaging counts for samples that were sequenced over multiple runs, and again removing any remaining singletons and doubletons. Only taxa classified as bacteria were kept for analysis; eukaryotic, archaeal, mitochondrial and chloroplast sequences were removed.

### Grouping gametophytes and taxonomy levels for biomass and population comparisons

Due to the smaller number of individuals within the AQ, CI, and CP populations, the comparison of network analysis across biomass outcomes was only performed with individuals from the LC population. A total of 308 individuals from the LC population were divided into one of four quantile groups based on their wet biomass weight at the time of harvest: Quantile 1 (≤ 63.92g, n = 77), Quantile 2 (> 63.92g and ≤ 125g, n = 78), Quantile 3 (> 125g and ≤ 211g, n = 76), and Quantile 4 (> 211g, n = 77). These biomass values represent that of diploid sporophytes grown on the farm. Recall that the crossing scheme used in this study crossed a single male gametophyte from the LC population with 500 female gametophytes across the AQ, CI, CP, and LC populations (see: ‘Production of gametophytes and cultivation of sporophytes’). Consequently, the microbial community of the corresponding female gametophyte for each sporophyte was analyzed. After running the bacterial classification pipeline described above, we conglomerated bacterial reads to four taxonomic levels (order, family, genus, and species) for all LC individuals. Analysis at several taxonomic levels was done to address the challenge of taxonomic resolution and classification uncertainty at higher levels (i.e. genus and species) and consider lower levels (i.e. order, and family) as proxies for ecological function [33,34]. For comparison of microbial networks across gametophytes from different kelp populations, individuals were grouped according to the geographic region (natural population) in which their parent sporophyte was collected: AQ (n = 64, 12 males and 52 females), CI (n = 57, 12 males and 45 females), CP (n = 69, 16 males and 53 females), and LC (n = 369, 54 males and 315 females). Because this comparison did not require the use of biomass data, we were able to source a larger number of individuals that were not grown on the farm. However, due to the increased number of taxa classified at the species level and the smaller number of samples, we were only able to run network analysis at the order and family levels.

### Quantification and visualization of co-occurrence network and hub taxa

Starting with network analysis across biomass outcomes, LC gametophytes (n = 308) were divided into four quantiles as described above. For each quantile, we randomly selected 50 individuals 100 times and constructed networks using the R package ‘SpiecEasi’ (version 1.1.0), which infers ecological associations in microbial communities [35]. The default settings for SpiecEasi with neighborhood selection (the Meinshausen and Bühlmann or “MB” method) were used [36]. The resulting representative network models were analyzed and graphed with the ‘igraph’ package (version 1.2.6) in R [37]. Networks were graphed with the Davidson-Harel layout algorithm (‘layout_with_dh’ function in igraph), which reduces edge crossing to produce a clean network [37,38]. For each network, the following network topology features were recorded: total nodes, total edges, number of positive edges, number of negative edges, ratio of positive to negative edges, average path length, heterogeneity, modularity, average degree per node, clustering coefficient, and hub score. Nodes represent unique taxa and edges are the significant co-occurrences between them. Positive edges indicate that connected taxa tend to be present together and negative edges indicate the opposite (i.e., if one is present in a community, the other is absent). Positive and negative edge information was also used to infer whether taxa of interest had competitive interactions with other taxa. The average path length considers the shortest edge path connecting each pair of nodes. Heterogeneity, the distribution of degrees or connections from each node, was calculated as described in Jacob et al. [39]. Modularity, the density of node connections compared to a randomly structured network, was measured with the Louvain method that maximizes the score for each community [40]. Hub score was calculated for the whole network without subsampling using Kleinberg’s centrality score, which ranges from 0 to 1 [41]. This method uses the adjacency matrix of a network, which represents the degrees connecting each node. Because this is an undirected graph, the hub score is the same as the authority score, and higher scores represent a greater number of edges (i.e., higher degrees) connected to each node. This pipeline was repeated with microbial networks classified at the order, family, genus, and species levels. For network analysis across gametophyte populations, we used the same pipeline and randomly selected 50 individuals 100 times from each population: AQ (n = 64), CI (n = 57), CP (n = 69), and LC (n = 369). Due to the higher complexity of these microbial networks and subsampling regime, we were unable to construct networks for the LC population at the genus and species levels. Therefore, we report only the network analysis done at the order and family levels across all four populations.

### Identifying network topology factors that predict sporophyte biomass

For network comparisons across biomass quantiles, we used gametophytes from the LC population (n = 308) and divided them into one of four quantile groups based on their wet biomass weight at the time of harvest: Quantile 1 (≤ 63.92g, n = 77), Quantile 2 (> 63.92g and ≤ 125g, n = 78), Quantile 3 (> 125g and ≤ 211g, n = 76), and Quantile 4 (> 211g, n = 77). We repeated analysis with bacteria conglomerated to the order, family, genus, and species levels. We constructed networks (described above) by randomly selecting 50 individuals 100 times from each quantile group. An ordered logistic regression model was estimated using the ‘polr’ command from the ‘MASS’ package (version 7.3.53) in R [42]. The model was first run using all non-multicollinear factors: total nodes, total edges, positive to negative edge ratio, average path length, modularity, average degree, heterogeneity, and clustering coefficient. Using the ‘regsubsets’ command from the ‘leaps’ package (version 3.1) in R we determined which network features are best associated with host biomass using co-occurrence networks generated from microbiomes classified at the following taxonomic levels: order, family, genus, and species. As stated earlier, analysis at several taxonomic levels was done to address the challenge of taxonomic resolution and classification uncertainty at higher levels (i.e. genus and species) and consider lower levels (i.e. order, and family) as proxies for ecological function [33,34]. Models were additionally confirmed for best fit factors using the ‘stepAIC’ command from MASS. In the case of a mismatch, which only occurred at the genus and species level, the simpler model was chosen. Log likelihoods were converted to odds ratios for ease of interpretation.

### Comparing network topology measures between populations

For network comparisons across populations, we analyzed gametophytes from four populations: AQ (n = 64), CI (n = 57), CP (n = 69), and LC (n = 369). Networks were constructed by randomly selecting 50 individuals 100 times from each population. Analysis was repeated with bacteria conglomerated to the order and family levels. We used a Kruskal Wallis test to determine if there was a significant difference overall across populations for the following topology measures: total nodes, total edges, ratio of positive to negative edges, average path length, modularity, average degree, heterogeneity, and clustering coefficient (S4 Table). Pairwise comparisons were tested for significant differences using a Wilcoxon test (S7–S10 Figs).

### Modeling community assembly patterns using the zeta diversity metric

To determine whether community assembly patterns differed between low- and high-biomass outcomes, we used zeta diversity to help determine the relative likelihoods of niche differentiated (non-random) and stochastic (random) processes of community assembly for kelp microbiomes found using either low- or high-biomass individuals. Due to the reduced number of individuals in the AQ, CI, and CP populations, this analysis was run on the biomass quantiles from the LC population alone. In order to model community assembly patterns and determine the degree to which microbial communities are randomly structured, we used the zeta diversity metric. This metric quantifies the number of species shared between any number of sites [18]. Zeta order refers to the number of sites being considered at a time when calculating their compositional overlap. As zeta order increases in size, the value of zeta diversity becomes increasingly influenced by more common species and the decline in the number of shared species can be modeled as an exponential or power-law regression. It has been found that the relative likelihoods of an exponential versus power-law model of zeta diversity is associated with the respective relatively likelihoods of a stochastic (random) versus niche-differentiated (non-random) model of community assembly [18]. For this study, the microbiome of each unique gametophyte is considered a “site”. Abundance counts were first converted to presence (1) and absence (0) scores. Zeta decline was modeled using the ‘zetadiv’ package (version 1.2.0) in R [43]. Comparison of AIC scores was used to determine best fitting model (exponential versus power-law regression) and more likely method of community assembly. Common species are shared between a higher number of sites while rare species are shared between fewer. Consequently, analysis was done for zeta orders 3, 5, 10, 20, and 50 at the species level to investigate the contribution of rare (lower zeta orders) versus common (higher zeta orders) species to compositional change. Analysis was also done at the class, order, family, and genus levels for zeta order 50 to determine if community assembly patterns differ between taxonomic levels.

## Results

### Network topology is associated with sporophyte biomass

**Fig 3:**
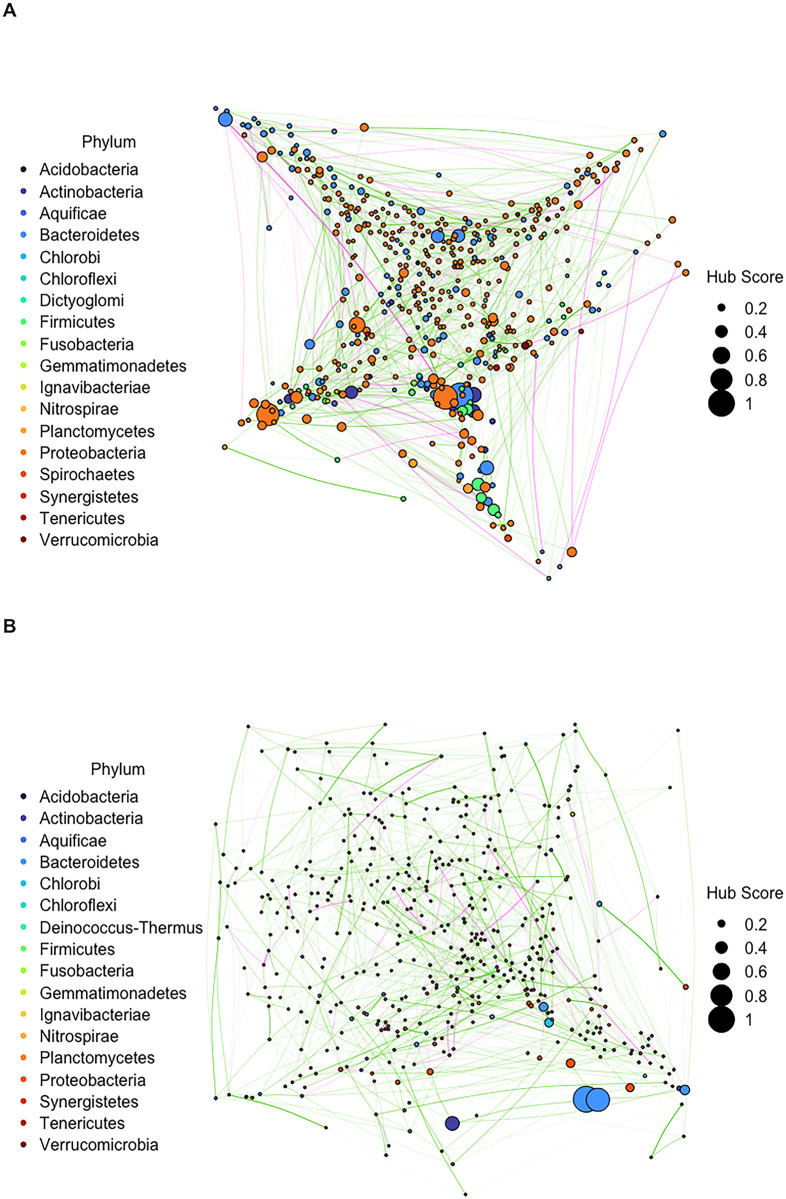
Co-occurrence networks of the microbial community classified at the genus level. Each node represents a unique genus. Node size represents the hub score and node color represents phylum membership. Edge opacity represents the strength of the link and edge color represents a positive (green) or negative (magenta) co-occurrence pattern. Microbial networks sampled from (A) low-biomass gametophytes (<63.92g, n = 77) and (B) high-biomass gametophytes (>211g, n = 77).

Using gametophytes from the Leo Carillo (LC) population (n = 308), we constructed co-occurrence networks of the microbial community with taxa classified at the order, family, genus, and species level (Figs 3 and S1). LC gametophytes were binned into one of four biomass quantile groups based on their sporophyte weight at the time of harvest. Network analysis was performed for each biomass group and topological measures of the co-occurrence networks of their associated microbiomes were recorded (S1 Table). To identify network topology factors that vary with biomass, we used a proportional odds logistic regression model. The best fit model for each taxonomic level included different topology factors (Tables 1 and S2). Clustering coefficient is associated with biomass across the order, family, genus, and species levels; however, its association with increased biomass changed across levels. At the order and species level, with a one unit increase in the clustering coefficient the odds of higher biomass was 3.52x10^3^ and 1.60x10^37^ more likely, respectively. At the family and genus levels, there was an opposite trend with higher biomass being 4.30x10^5^ and 4.07x10^7^ times less likely, respectively. Positive to negative edge ratio was also associated with biomass at the order, family, and genus levels: with each one unit increase (i.e. a higher proportion of positive associations between taxa) the odds of higher biomass was 1.04, 1.22, and 1.22 times more likely, respectively. Increased heterogeneity and lower modularity were associated with higher biomass at the order and family levels. For each one unit increase in heterogeneity, increased biomass is 1.00x10^9^ and 6.34x10^9^ times more likely. For each one unit increase in modularity, the odds of increased biomass was 1.97x10^7^ and 4.50x10^9^ times less likely. Finally, for average path length at the order level with each one unit increase the odds of increased biomass was 1.75 times less likely.

**Table 1:** Odds ratio values for network topology factors used in POLR models.

	Clustering Coefficient	Positive to Negative Edge Ratio	Heterogeneity	Modularity	Average Path Length
**Order**	3.52x10^3^	1.04	1.00x10^9^	5.07x10^-8 a^	5.73x10^-1 a^
**Family**	2.33x10^-6^ ^a^	1.22	6.34x10^9^	2.22x10^-10^ ^a^	
**Genus**	2.46x10^-8^ ^a^	4.65	NS		
**Species**	1.60x10^37^				

Summary of odds ratio values for network topology factors (p < 0.01) used in proportional odds logistic regression (POLR) models. Separate models were built for the order, family, genus, and species taxonomic levels. Factors to include for each model was determined by best fit and blanks indicate that a factor was not used in the model. (For example, at the species level: Biomass Quantile ~ Clustering Coefficient.) ‘NS’ signifies that although used in the model, the factor was not significantly associated with biomass

^a^The odds ratio values, which are recorded in this table, can be challenging to interpret. For ease of interpretation, the reciprocal for values with negative exponents is calculated to represent how “less likely” the odds of increased biomass is with each one unit increase in the corresponding network topology factor and is referenced this way in the main text. Values with positive exponents are interpreted as that much “more likely” to have increased biomass with each one unit increase in the corresponding network topology factor.

### Microbial communities from gametophytes that become low- or high-biomass sporophytes have unique hub taxa

From the network analysis described above we also calculated hub scores for each taxa and identified those with the highest scores (Fig 3, Table 2). Here we define hub taxa as those that had a score of at least 0.5 and we report those from the order, family, genus, and species levels (Table 2). Microbial communities from gametophytes that became low-biomass sporophytes (<63.92g) had the following hub taxa (score followed in parentheses): orders Frankiales (1) and Kineosporiales (0.89); families Veillonellaceae (1), Archangiaceae (0.99), Burkholderiaceae (0.89), and Clostridiaceae 1 (0.85); genera *Marixanthomonas* (1), *Magnetococcus* (0.91), *Epibacterium* (0.84), *alpha proteobacterium PWB3*(0.57), and *Collinsella* (0.53); species *Kordiimonas lacus* (1), *Methylosinus trichosporium* (0.87), *alpha proteobacterium SAORIC-651* (0.74), *Stappia taiwanensis* (0.62), and *marine bacterium VA011* (0.55). In general, high-biomass sporophytes (>211g) had fewer hub taxa in the gametophyte microbial communities. High-biomass hub taxa were orders Desulfovibrionales (1), Nitrospinales (0.99); families Magnetococcaceae (1), Beijerinckiaceae (0.95), and Holosporaceae (0.81); genera *Wenyingzhuangia* (1) and *Pedobacter* (0.89); and species *mucus bacterium 80* (1) and *Marinomonas brasilensis* (0.59).

**Table 2:** Hub taxa by biomass group.

Taxonomic Level	Taxa	Hub Score	Biomass Group
**Order**	**Desulfovibrionales**	**1**	**High**
**Order**	**Nitrospinales**	**0.99**	**High**
**Family**	**Magnetococcaceae**	**1**	**High**
**Family**	**Beijerinckiaceae**	**0.95**	**High**
**Family**	**Holosporaceae**	**0.81**	**High**
**Genus**	**Wenyingzhuangia**	**1**	**High**
**Genus**	**Pedobacter**	**0.89**	**High**
**Species**	**mucus bacterium 80**	**1**	**High**
**Species**	**Marinomonas brasilensis**	**0.59**	**High**
**Order**	**Frankiales**	**1**	**Low**
**Order**	**Kineosporiales**	**0.89**	**Low**
**Family**	**Veillonellaceae**	**1**	**Low**
**Family**	**Archangiaceae**	**0.99**	**Low**
**Family**	**Burkholderiaceae**	**0.89**	**Low**
**Family**	**Clostridiaceae 1**	**0.85**	**Low**
**Genus**	**Marixanthomonas**	**1**	**Low**
**Genus**	**Magnetococcus**	**0.91**	**Low**
**Genus**	**Epibacterium**	**0.84**	**Low**
**Genus**	**alpha proteobacterium PWB3a**	**0.57**	**Low**
**Genus**	**Collinsella**	**0.53**	**Low**
**Species**	**Kordiimonas lacus**	**1**	**Low**
**Species**	**Methylosinus trichosporium**	**0.87**	**Low**
**Species**	**alpha proteobacterium SAORIC-651**	**0.74**	**Low**
**Species**	**Stappia taiwanensis**	**0.62**	**Low**
**Species**	**marine bacterium VA011**	**0.55**	**Low**

Hub taxa with a Kleinberg’s centrality score of over 0.5. *M*. *pyrifera* gametophytes from the Leo Carillo population were binned into biomass groups based on their sporophyte weight at the time of harvest. Representative networks were generated for the microbial communities of each biomass group. Taxa were then given a score to quantify their role as a hub taxa. Taxa from the genus and species levels that scored over 0.5 are recorded here. Biomass groups: Low (<63.92g, n = 77) and High (>211g, n = 77). Taxa names are listed as the direct outputs from the metaxa2 classification pipeline with the SILVA 128 database.

^a^The SILVA taxonomy database is manually curated and shown to have guide tree errors [44]. This species appears to have been incorrectly classified as a genus.

### Candidate growth-promoting taxa co-occurs with hub microbes of gametophytes that become high-biomass sporophytes

In a previous study, we found that bacteria from the genus *Mesorhizobium* is associated with increased biomass of *M*. *pyrifera* and therefore a prime candidate for a growth-promoting inoculant [22]. Using the representative networks constructed for this study, we investigated the positive and negative associations *Mesorhizobium* has with other taxa in the microbial community of *M*. *pyrifera* gametophyte germplasm cultures. We found that *Mesorhizobium* co-occurs with *Wenyingzhuangia* and *Pedobacter*, which had the two highest hub scores for gametophytes that become high-biomass sporophytes. We also found that *Mesorhizobium* has negative co-occurrence values with *Aquamarina*, *Sneathiella*, *Pseudohaliea*, and *Saccharospirillum*.

### Network topology and hub taxa differs between gametophyte populations

**Fig 4:**
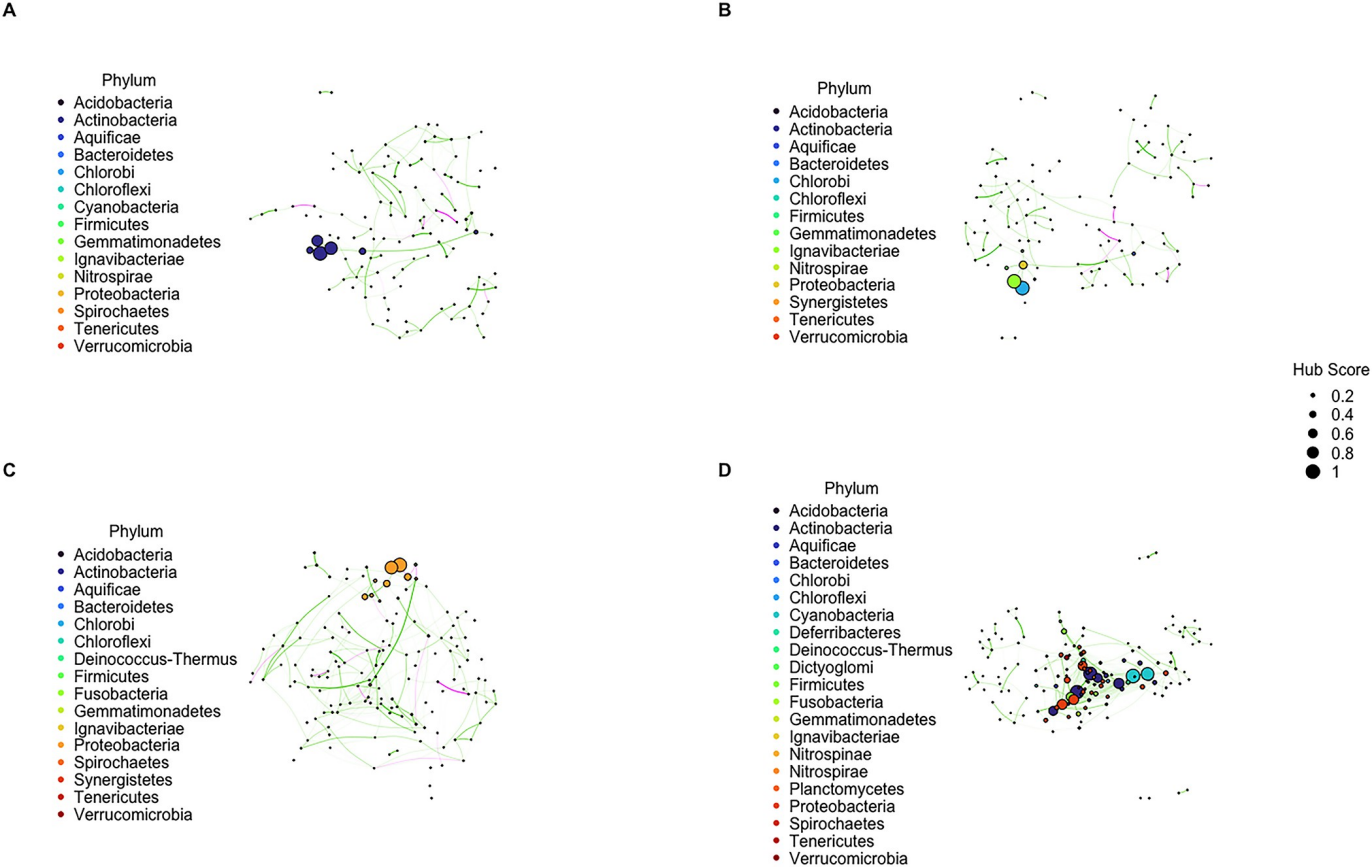
Co-occurrence networks of the microbial community classified at the family level. Each node represents a unique family. Node size represents the hub score and node color represents phylum membership. Edge opacity represents the strength of the link and edge color represents a positive (green) or negative (magenta) co-occurrence pattern. Microbial networks sampled from four populations: (A) AQ, (B) CI, (C) CP, and (D) LC.

Using gametophytes from all four populations (AQ, CI, CP, and LC), we constructed co-occurrence networks of the microbial community with taxa classified at the order and family levels (Figs 4 and S6). We investigated whether there was a significant difference across populations for the following network topology measures: total nodes, total edges, ratio of positive to negative edges, average path length, modularity, average degree, heterogeneity, and clustering coefficient (S4 and S5 Figs). We found that there was a significant difference overall for all topology measures. Pairwise comparisons were significant for all combinations for total nodes, total edges, average degree, and clustering coefficient. For the remaining measures, most combinations were significantly different except for the following: ratio of positive to negative edges and modularity for the AQ and CI populations, average path length for the CI and CP populations, and heterogeneity for the AQ and CP populations. In addition to differences among network topology measures, we also found that the four populations had distinct hub taxa. In general, the LC population had the greatest number of hub taxa with a score over 0.5. Of those identified, only two taxa overlapped between populations: Chthoniobacterales was shared between the AQ and CP populations and Cryptosporangiaceae was shared between the AQ and LC populations (S3 Table).

### Community assembly of gametophyte microbial community is niche-driven across biomass outcomes

Zeta diversity, the number of shared species between three or more sites, was used to model community assembly patterns and determine whether they are driven by stochastic (random) or niche-driven (non-random) mechanisms. Zeta order refers to the number of sites included in this measure. Here, sites refer to gametophyte microbiome samples. To understand the contribution of rare and common taxa to compositional change we ran zeta diversity analysis at zeta orders 3, 5, 10, 20, and 50. We found that for gametophytes that became high-biomass sporophytes, zeta diversity decline of microbial communities follow a power-law regression of zeta diversity decline for all zeta orders (S2 Fig). To determine whether community assembly patterns vary across taxonomic levels, we additionally ran analysis with zeta order 50 at the class, order, family, and genus levels (S3 Fig). All taxonomic levels demonstrated niche-driven assembly patterns across biomass outcomes.

## Discussion

Analysis of the microbial co-occurrence network topology in gametophytes cultures across biomass outcomes revealed that several features are significantly associated with sporophyte yield. Clustering coefficient and the ratio of positive to negative edges were identified as significant factors associated with sporophyte biomass when looking at gametophyte microbial networks classified at the class, order, family, and genus levels. At the species level, larger clustering coefficient values, which are associated with highly complex communities and strong microbe-microbe interactions [45], have a profoundly high likelihood of increased biomass. This suggests that densely connected subnetworks are associated with improved growth in *M*. *pyrifera*. Although topological analysis does not offer insight on the mechanisms behind this impact, higher clustering coefficients may suggest greater cooperation [46] that can benefit the host. Likewise, a higher ratio of positive to negative edges associated with increased biomass suggests less competition between taxa that could detract from host health and performance. At the order and family levels, increased heterogeneity, indicating more variation in the number of connecting edges per node, suggests that when the edge connections of a network are concentrated on a small number of taxa there is a growth benefit to the host. In other words, this may indicate that having few hub taxa (with dense connections to other members of the community) that dominate associations across the network is beneficial.

We confirmed that hub taxa are different for *M*. *pyrifera* gametophytes that become low- and high-biomass sporophytes. It is important to note that hub nodes are not necessarily the most abundant taxa, and that hub nodes identified in this paper do not overlap with the most abundant microbes of high-biomass sporophytes identified in our previous study [22]. Although not necessarily the most abundant, hub microbes impact the colonization and abundance of other bacteria [17]. They may also impact host physiology, including host metabolism, which indirectly impacts what microbial species are present [17]. It is possible that hub microbes from low-biomass hosts may be inefficient at recruiting bacteria that provide the greatest growth benefit to the host. Consequently, future studies should investigate whether this relationship may be exploited to recruit beneficial microbes at the early stage of seaweeds and increase growth. In particular, the addition of taxa from the genera *Wenyingzhuangia* and *Pedobacter* or the addition of species *mucus bacterium 80* and *Marinomonas brasilensis* are promising directions to test whether inoculation at the early life stage of *M*. *pyrifera* will recruit other beneficial bacteria and induce a growth-promoting benefit. Future work may also focus on isolating and sequencing these taxa to gain insight on their functional capability and mechanisms for regulating microbe-microbe and microbe-host interactions overall [6]. While we did not analyze the correlation between hub score and abundance in this study, this would be a useful metric to include for future work. We discovered that *Mesorhizobium*, which is a prime candidate for growth-promoting inoculants in *M*. *pyrifera* [22], does not have negative associations with *Wenyingzhuangia* nor *Pedobacter*. This suggests that if bacteria from these three genera were included in a growth-promoting inoculant they would not compete with each other and perhaps even provide a synergistic effect. This is a promising finding given that the perturbance and removal of hub taxa can have negative cascading effects throughout a microbial community and decrease stability overall. The genera that *Mesorhizobium* does not co-occur with (*Aquamarina*, *Sneathiella*, *Pseudohaliea*, and *Saccharospirillum*) are not hub taxa; further investigation is needed to determine if taxa from these genera would directly compete with, or disrupt the efficacy of, a *Mesorhizobium* inoculation.

In line with previous findings that microbial community diversity significantly differs across populations [22], we found that network dynamics similarly vary by population. This is likely a consequence of diverse taxa inhabiting *M*. *pyrifera* individuals from different populations, perhaps driven by genetic diversity of kelp gametophytes. Of particular interest, even though network variations across biomass outcomes were only analyzed in the LC population, it is possible that gametophytes from other populations will respond positively to inoculation with hub microbes of high-biomass LC gametophytes given that a previous study demonstrated that *M*. *pyrifera* gametophytes from San Diego had increased length and abundance when grown in different microbial treatments of seawater from Catalina [47]. While network topology analysis increases our understanding of the structural traits associated with increased biomass, it will be more insightful to layer this work with other data types including those from genomics and metabolomics to infer functional mechanisms impacting host growth [10].

Zeta diversity analysis revealed that the microbial community assembles in a niche-driven manner when conglomerated to the class, order, family, genus, and species levels and that this is consistent across all biomass outcomes. At the species level, rare and common species similarly contribute to this assembly pattern. Together, this suggests that the community is competitively structured and that assembly patterns are not a driving factor in the difference between biomass yields for *M*. *pyrifera* cultivars. This may make the design and introduction of growth-promoting inoculants more challenging. Inoculants will have to be designed in a way that does not compete with established niches so that it can persist in the context of the native microbial community.

It is important to acknowledge that findings from this study are limited by several factors that should be addressed in future studies. The database used for bacterial classification, SILVA 128, is not the most recent available. This version was chosen to facilitate comparison between findings from this and a previous study [22]. Before applying the findings from this work to seaweed aquaculture, it would be beneficial to re-run analysis with more recent, and perhaps several, databases to confirm whether the same trends exist. The dataset used in this study faces a dimensionality problem where the number of taxa is often greater than the number of samples (S5 Table). This can result in poor network recovery and a high false positive rate. Although SpieacEasi is able to construct networks with fewer samples than taxa and performs well compared to other tools [48], the findings presented here are likely not representative of full network recovery. Future iterations of this work should either apply stronger filters to focus on taxa of interest or incorporate more samples. Furthermore, only one network construction method, SpiecEasi, was used. In order to more fully understand how network features are associated with biomass yields it will be important to explore alternate network construction methods, such as the SpiecEasi ‘glasso’ method or an entirely different tool such as SparCC [49]. Lastly, the Davidson-Harel layout algorithm used in this study to construct microbial networks is not appropriate for deriving biological interpretations from node location, as the graphs are constructed to reduce edge crossing. Future work should consider alternate network layout algorithms that enable biological interpretation of node placement to gain deeper insight from network analysis.

In conclusion, we analyzed the network dynamics and community assembly patterns of microbial communities for cultivated *M*. *pyrifera* gametophytes and compared these characteristics with sporophyte performance to ultimately identify features associated with increased biomass. We found that the network dynamics and hub taxa of microbial communities at the gametophyte stage may be a driving force in biomass outcomes at the sporophyte stage. In addition, we found that microbial communities assemble in a niche-driven manner across all biomass outcomes. When designing inoculants to increase the biomass yield of *M*. *pyrifera* cultivars, avoiding competition with hub taxa identified here may increase long-term efficacy. Introduction of desired hub taxa at the gametophyte stage can also induce the recruitment of other beneficial bacteria and shape the overall community in a more precise manner. There are several exciting opportunities for future research to help us better understand microbe-microbe interactions and their impact on the host, such as genome sequencing of hub taxa to elucidate functional pathways and genome-wide association studies to identify genetic factors of *M*. *pyrifera* that impact recruitment of these taxa. Incorporating analysis of the host genome is particularly exciting for growth-promoting applications discussed here as the impact that host genotype can have on the overall microbial community are strongest if focused on hub microbes [17]. Finally, inoculation trials will need to be performed to track long-term efficacy, change in biomass outcomes, and impact on network structure. Altogether, this is a helpful study that will support the use of growth-promoting microbial inoculants in *M*. *pyrifera* cultivars and seaweed aquaculture more broadly.

## Supporting information

S1 Fig Co-occurrence networks of the microbial community sampled from LC gametophytes. Co-occurrence networks classified at the (A-B) order, (C-D) family, and (E-F) species levels. Each node represents a unique taxa. Node size represents the hub score and node color represents phylum membership. Edge opacity represents the strength of the link and edge color represents a positive (green) or negative (magenta) co-occurrence pattern. (A, C, E) Microbial network sampled from low-biomass gametophytes (<63.92g, n = 77). (B, D, F) Microbial network sampled from high-biomass gametophytes (>211g, n = 77).
(DOCX)

S2 Fig Zeta diversity graphs for zeta order 3, 5, 10, 20, and 50 at the species level. Zeta diversity decline, decline ratio, exponential and power-law regression graphs. For zeta orders (A) 3, (B) 5, (C) 10, (D) 20, and (E) 50. Results shown are for gametophytes that became high-biomass sporophytes. Columns from left to right: Zeta diversity decline representing the number of shared species (Zeta diversity, y-axis) against zeta order; Ratio of zeta diversity decline, also called the “retention rate curve” that plots the zeta ratios (Zi+1/ Zi) against Zi; zeta decline curves fitted against exponential and power-law regressions. AIC scores of the two models confirmed that power-law regression is a better fit for all variations.
(DOCX)

S3 Fig Zeta diversity graphs for zeta order 50 at order, family, genus, and species levels. Zeta diversity decline, decline ratio, exponential and power-law regression graphs. For zeta order 50 at taxonomic levels (A) order, (B) family, (C) genus, and (D) species. Results shown are for gametophytes that became high-biomass sporophytes. Columns from left to right: Zeta diversity decline representing the number of shared species (Zeta diversity, y-axis) against zeta order; Ratio of zeta diversity decline, also called the “retention rate curve” that plots the zeta ratios (Zi+1/ Zi) against Zi; zeta decline curves fitted against exponential and power-law regressions. AIC scores of the two models confirmed that power-law regression is a better fit for all variations.
(DOCX)

S4 Fig Box plots of network topology factors at the order level. Box plots of (A) total nodes, (B) total edges, (C) positive to negative edge ratio, (D) average path length, (E) modularity, (F) average degree, (G) heterogeneity, and (H) clustering coefficient for all populations (AQ, CI, CP, and LC) with bacteria classified at the order level. Pairwise significance was tested with the Wilcoxon test: ns: not significant, *: p < = 0.05, **: p < = 0.01, ***: p< = 0.001, ****: p < = 0.0001.
(DOCX)

S5 Fig Box plots of network topology factors at the family level. Box plots of (A) total nodes, (B) total edges, (C) positive to negative edge ratio, (D) average path length, (E) modularity, (F) average degree, (G) heterogeneity, and (H) clustering coefficient for all populations (AQ, CI, CP, and LC) with bacteria classified at the family level. Pairwise significance was tested with the Wilcoxon test: ns: not significant, *: p < = 0.05, **: p < = 0.01, ***: p< = 0.001, ****: p < = 0.0001.
(TIFF)

S6 Fig Co-occurrence networks of the microbial community classified at the order level. Each node represents a unique taxa. Node size represents the hub score and node color represents phylum membership. Edge opacity represents the strength of the link and edge color represents a positive (green) or negative (magenta) co-occurrence pattern. Microbial networks sampled from four populations: (A) AQ, (B) CI, (C) CP, and (D) LC.
(DOCX)

S7 Fig Box plots of network topology factors by biomass quantile at the order level. Box plots of (A) total nodes, (B) total edges, (C) positive to negative edge ratio, (D) average path length, (E) modularity, (F) average degree, (G) heterogeneity, and (H) clustering coefficient for all biomass quantiles (Q1, Q2, Q3, Q4) with bacteria classified at the order level. Pairwise significance was tested with the Wilcoxon test: ns: not significant, *: p < = 0.05, **: p < = 0.01, ***: p< = 0.001, ****: p < = 0.0001.
(DOCX)

S8 Fig Box plots of network topology factors by biomass quantile at the family level. Box plots of (A) total nodes, (B) total edges, (C) positive to negative edge ratio, (D) average path length, (E) modularity, (F) average degree, (G) heterogeneity, and (H) clustering coefficient for all biomass quantiles (Q1, Q2, Q3, Q4) with bacteria classified at the family level. Pairwise significance was tested with the Wilcoxon test: ns: not significant, *: p < = 0.05, **: p < = 0.01, ***: p< = 0.001, ****: p < = 0.0001.
(DOCX)

S9 Fig Box plots of network topology factors by biomass quantile at the genus level. Box plots of (A) total nodes, (B) total edges, (C) positive to negative edge ratio, (D) average path length, (E) modularity, (F) average degree, (G) heterogeneity, and (H) clustering coefficient for all biomass quantiles (Q1, Q2, Q3, Q4) with bacteria classified at the genus level. Pairwise significance was tested with the Wilcoxon test: ns: not significant, *: p < = 0.05, **: p < = 0.01, ***: p< = 0.001, ****: p < = 0.0001.
(DOCX)

S10 Fig Box plots of network topology factors by biomass quantile at the species level. Box plots of (A) total nodes, (B) total edges, (C) positive to negative edge ratio, (D) average path length, (E) modularity, (F) average degree, (G) heterogeneity, and (H) clustering coefficient for all biomass quantiles (Q1, Q2, Q3, Q4) with bacteria classified at the species level. Pairwise significance was tested with the Wilcoxon test: ns: not significant, *: p < = 0.05, **: p < = 0.01, ***: p< = 0.001, ****: p < = 0.0001.
(DOCX)

S1 Table Summary of network topology factors. Network topology factors recorded for LC gametophytes (n = 308) with bacteria classified at four taxonomic levels: order, family, genus, and species. LC gametophytes were divided into four biomass quantiles and a summary of all data is presented here. For each taxonomic level, we randomly sampled 50 individuals from each quantile 100 times to create representative networks. Values have been rounded to four significant figures. (*) Used in regression model.
(DOCX)

S2 Table Summary of p-value and odds ratio values. P-value and odds ratio values for network topology factors used in proportional odds logistic regression (POLR) model. Models were as follows: (Order) Biomass Quantile ~ Positive to Negative Edge Ratio + Average Path Length + Modularity + Heterogeneity + Clustering Coefficient, (Family) Biomass Quantile ~ Positive to Negative Edge Ratio + Modularity + Heterogeneity + Clustering Coefficient, (Genus) Biomass Quantile ~ Positive to Negative Edge Ratio + Heterogeneity + Clustering Coefficient, and (Species) Biomass Quantile ~ Clustering Coefficient.
(DOCX)

S3 Table Hub taxa by population. Hub taxa with a Kleinberg’s centrality score of over 0.5. *M*. *pyrifera* gametophytes from all four populations (AQ, CI, CP, and LC). Representative networks were generated for the microbial communities of each population. Taxa were then given a score to quantify their role as a hub taxa. Taxa from the order and family levels that scored over 0.5 are recorded here. *,+ denotes hub taxa found in more than one population with a score over 0.5.
(DOCX)

S4 Table Significant differences between network features across biomass outcomes. Resulting p-values for Kruskal-Wallis rank sum test comparing network features across all biomass quantiles. Results recorded for networks built with bacteria classified at the order, family, genus, and species levels.
(DOCX)

S5 Table Sample size and number of taxa. Number of samples and number of taxa that were used in network construction for all four biomass quantiles from the LC population.
(DOCX)
