# Supplementary material for: Investigating the relationship between microbial network features of giant kelp “seedbank” cultures and subsequent farm performance
Source: PLoS One. 2024 Mar 27;19(3):e0295740. doi: 10.1371/journal.pone.0295740 (PMC10971754; doi:10.1371/journal.pone.0295740)
Supplement: S1 Fig — Co-occurrence networks classified at the (A-B) order, (C-D) family, and (E-F) species levels. Each node represents a unique taxa. Node size represents the hub score and node color represents phylum membership. Edge opacity represents the strength of the link and edge color represents a positive (green) or negative (magenta) co-occurrence pattern. (A, C, E) Microbial network sampled from low-biomass gametophytes (<63.92g, n = 77). (B, D, F) Microbial network sampled from high-biomass gametophytes (>211g, n = 77). (DOCX) [file pone.0295740.s001.docx]

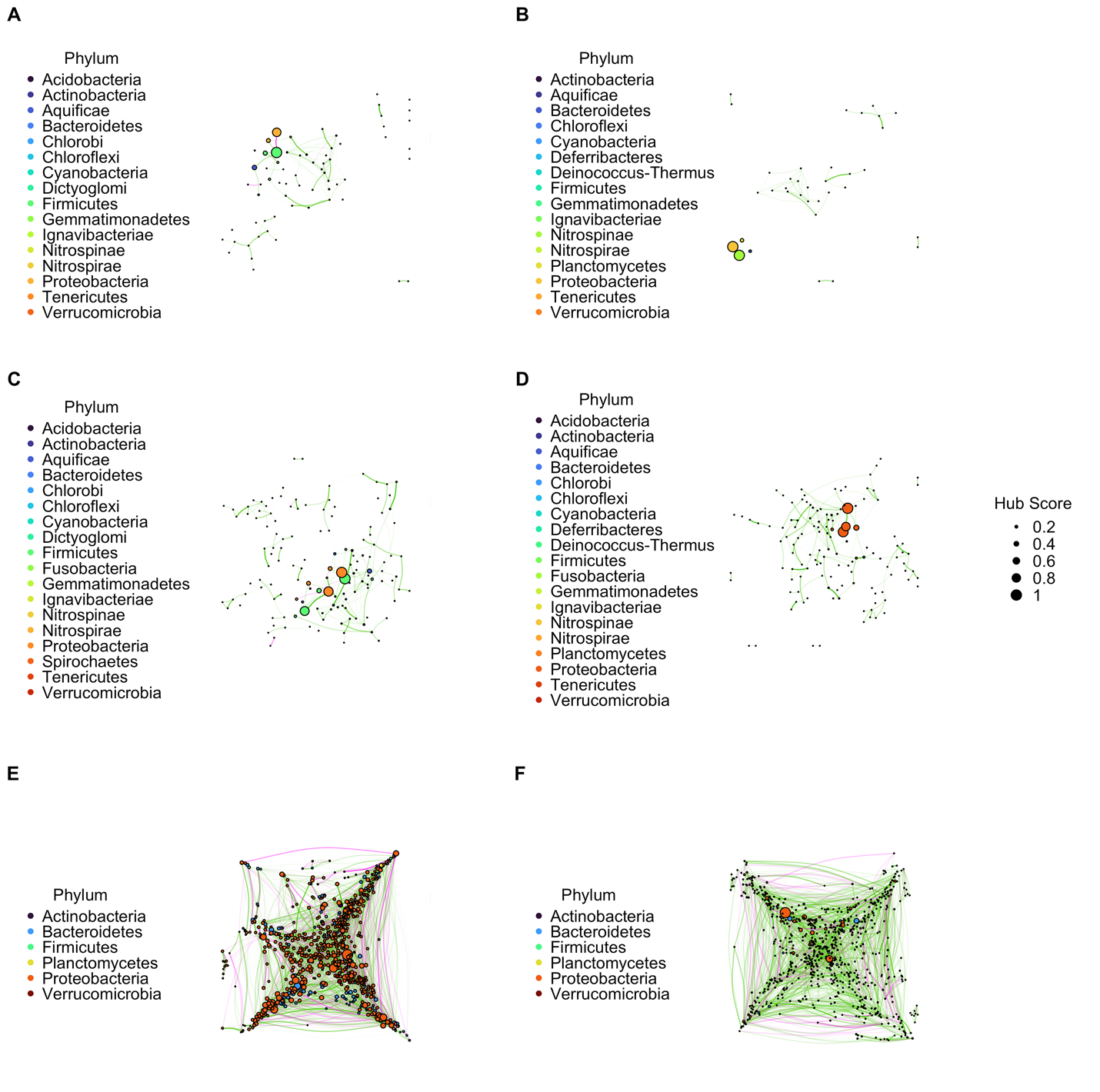


**S1 Fig. Co-occurrence networks of the microbial community sampled from LC gametophytes.** Co-occurrence networks classified at the (A-B) order, (C-D) family, and (E-F) species levels. Each node represents a unique taxa. Node size represents the hub score and node color represents phylum membership. Edge opacity represents the strength of the link and edge color represents a positive (green) or negative (magenta) co-occurrence pattern. (A, C, E) Microbial network sampled from low-biomass gametophytes (<63.92g, n = 77). (B, D, F) Microbial network sampled from high-biomass gametophytes (>211g, n = 77).
