# Supplementary material for: Investigating the relationship between microbial network features of giant kelp “seedbank” cultures and subsequent farm performance
Source: PLoS One. 2024 Mar 27;19(3):e0295740. doi: 10.1371/journal.pone.0295740 (PMC10971754; doi:10.1371/journal.pone.0295740)
Supplement: S2 Fig — Zeta diversity decline, decline ratio, exponential and power-law regression graphs. For zeta orders (A) 3, (B) 5, (C) 10, (D) 20, and (E) 50. Results shown are for gametophytes that became high-biomass sporophytes. Columns from left to right: Zeta diversity decline representing the number of shared species (Zeta diversity, y-axis) against zeta order; Ratio of zeta diversity decline, also called the “retention rate curve” that plots the zeta ratios (Zi+1/ Zi) against Zi; zeta decline curves fitted against exponential and power-law regressions. AIC scores of the two models confirmed that power-law regression is a better fit for all variations. (DOCX) [file pone.0295740.s002.docx]

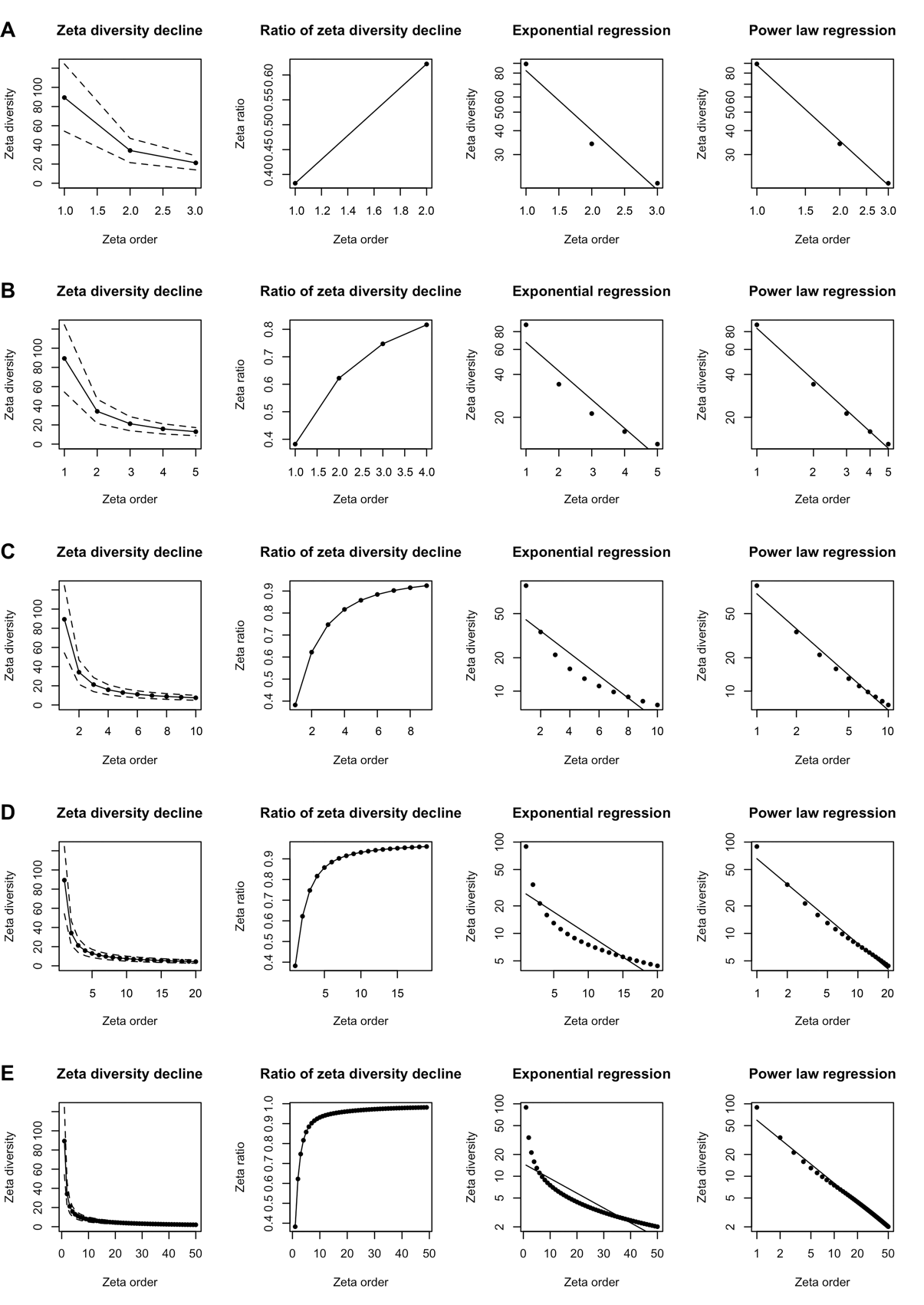


**S2 Fig. Zeta diversity graphs for zeta order 3, 5, 10, 20, and 50 at the species level.** Zeta diversity decline, decline ratio, exponential and power-law regression graphs. For zeta orders (A) 3, (B) 5, (C) 10, (D) 20, and (E) 50. Results shown are for gametophytes that became high-biomass sporophytes. Columns from left to right: Zeta diversity decline representing the number of shared species (Zeta diversity, y-axis) against zeta order; Ratio of zeta diversity decline, also called the “retention rate curve” that plots the zeta ratios (Zi+1/ Zi) against Zi; zeta decline curves fitted against exponential and power-law regressions. AIC scores of the two models confirmed that power-law regression is a better fit for all variations.
