# Supplementary material for: Investigating the relationship between microbial network features of giant kelp “seedbank” cultures and subsequent farm performance
Source: PLoS One. 2024 Mar 27;19(3):e0295740. doi: 10.1371/journal.pone.0295740 (PMC10971754; doi:10.1371/journal.pone.0295740)
Supplement: S6 Fig — Each node represents a unique taxa. Node size represents the hub score and node color represents phylum membership. Edge opacity represents the strength of the link and edge color represents a positive (green) or negative (magenta) co-occurrence pattern. Microbial networks sampled from four populations: (A) AQ, (B) CI, (C) CP, and (D) LC. (DOCX) [file pone.0295740.s006.docx]

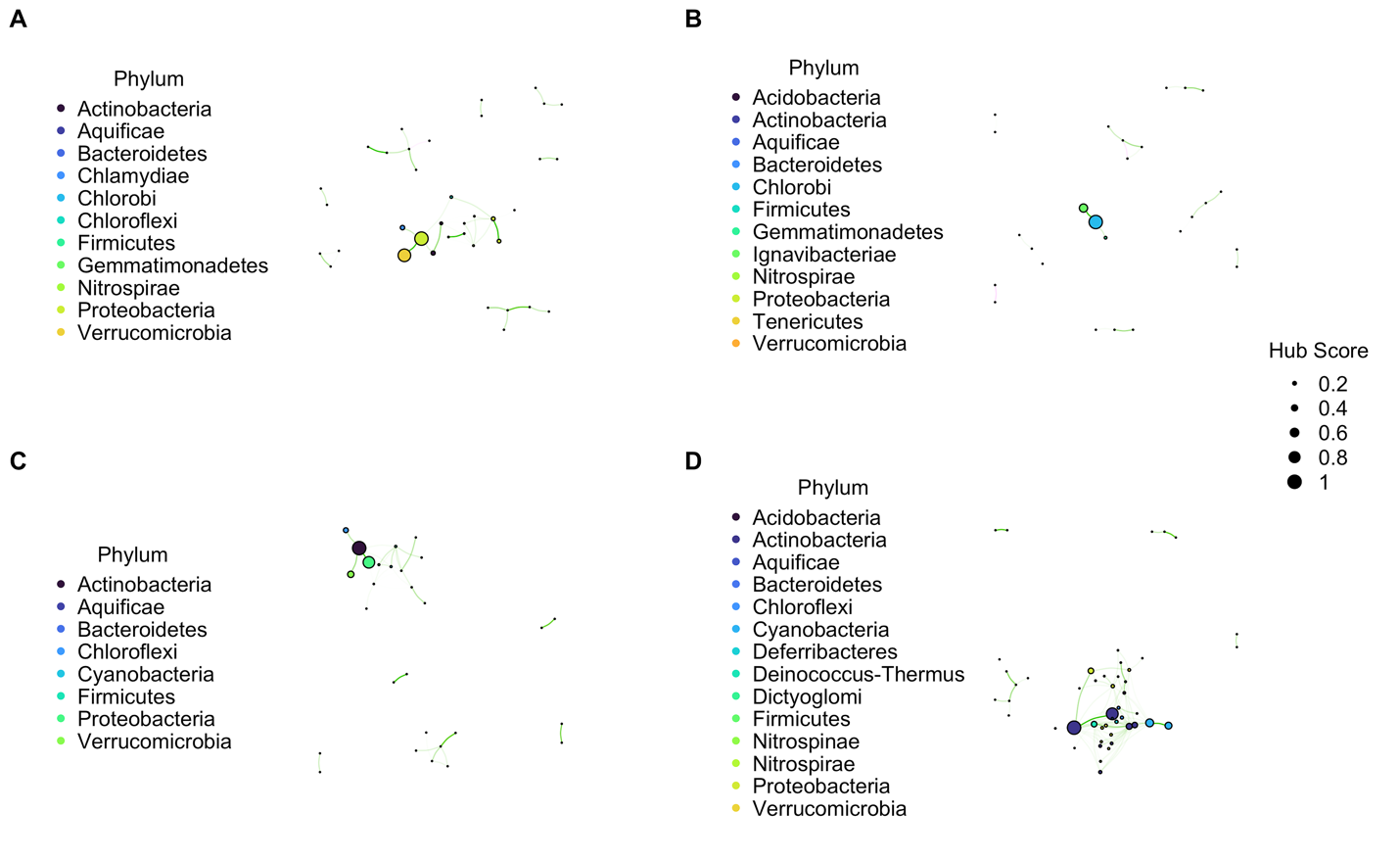


**S6 Fig. Co-occurrence networks of the microbial community classified at the order level.** Each node represents a unique taxa. Node size represents the hub score and node color represents phylum membership. Edge opacity represents the strength of the link and edge color represents a positive (green) or negative (magenta) co-occurrence pattern. Microbial networks sampled from four populations: (A) AQ, (B) CI, (C) CP, and (D) LC.
