# Supplementary material for: Investigating the relationship between microbial network features of giant kelp “seedbank” cultures and subsequent farm performance
Source: PLoS One. 2024 Mar 27;19(3):e0295740. doi: 10.1371/journal.pone.0295740 (PMC10971754; doi:10.1371/journal.pone.0295740)
Supplement: S8 Fig — Box plots of (A) total nodes, (B) total edges, (C) positive to negative edge ratio, (D) average path length, (E) modularity, (F) average degree, (G) heterogeneity, and (H) clustering coefficient for all biomass quantiles (Q1, Q2, Q3, Q4) with bacteria classified at the family level. Pairwise significance was tested with the Wilcoxon test: ns: not significant, *: p < = 0.05, **: p < = 0.01, ***: p< = 0.001, ****: p < = 0.0001. (DOCX) [file pone.0295740.s008.docx]

**
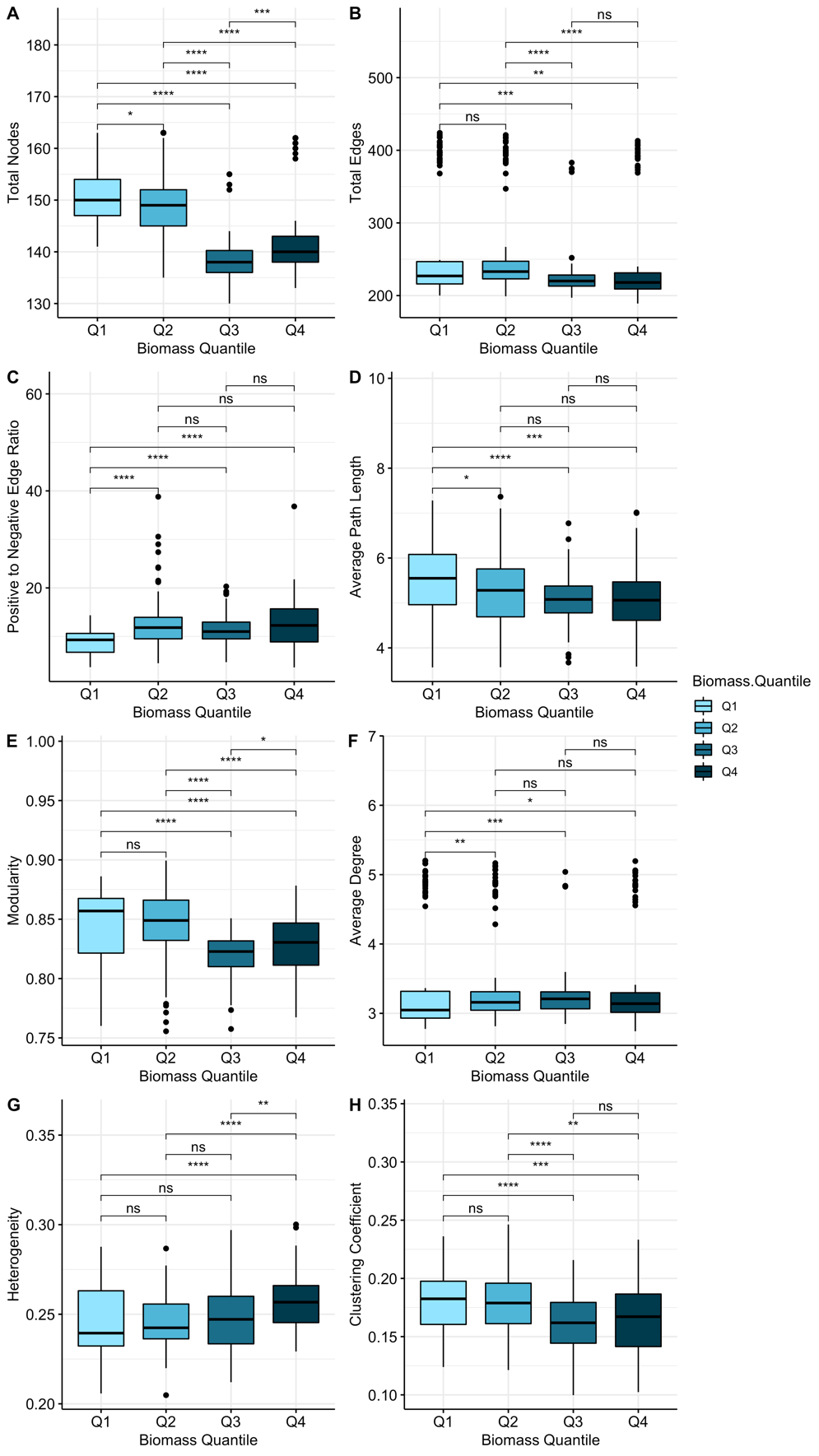
**

**S8 Fig. Box plots of network topology factors by biomass quantile at the family level.** Box plots of (A) total nodes, (B) total edges, (C) positive to negative edge ratio, (D) average path length, (E) modularity, (F) average degree, (G) heterogeneity, and (H) clustering coefficient for all biomass quantiles (Q1, Q2, Q3, Q4) with bacteria classified at the family level. Pairwise significance was tested with the Wilcoxon test: ns: not significant, *: p <= 0.05, **: p <= 0.01, ***: p<=0.001, ****: p <= 0.0001.
