# Supplementary material for: Investigating the relationship between microbial network features of giant kelp “seedbank” cultures and subsequent farm performance
Source: PLoS One. 2024 Mar 27;19(3):e0295740. doi: 10.1371/journal.pone.0295740 (PMC10971754; doi:10.1371/journal.pone.0295740)
Supplement: S10 Fig — Box plots of (A) total nodes, (B) total edges, (C) positive to negative edge ratio, (D) average path length, (E) modularity, (F) average degree, (G) heterogeneity, and (H) clustering coefficient for all biomass quantiles (Q1, Q2, Q3, Q4) with bacteria classified at the species level. Pairwise significance was tested with the Wilcoxon test: ns: not significant, *: p < = 0.05, **: p < = 0.01, ***: p< = 0.001, ****: p < = 0.0001. (DOCX) [file pone.0295740.s010.docx]

**
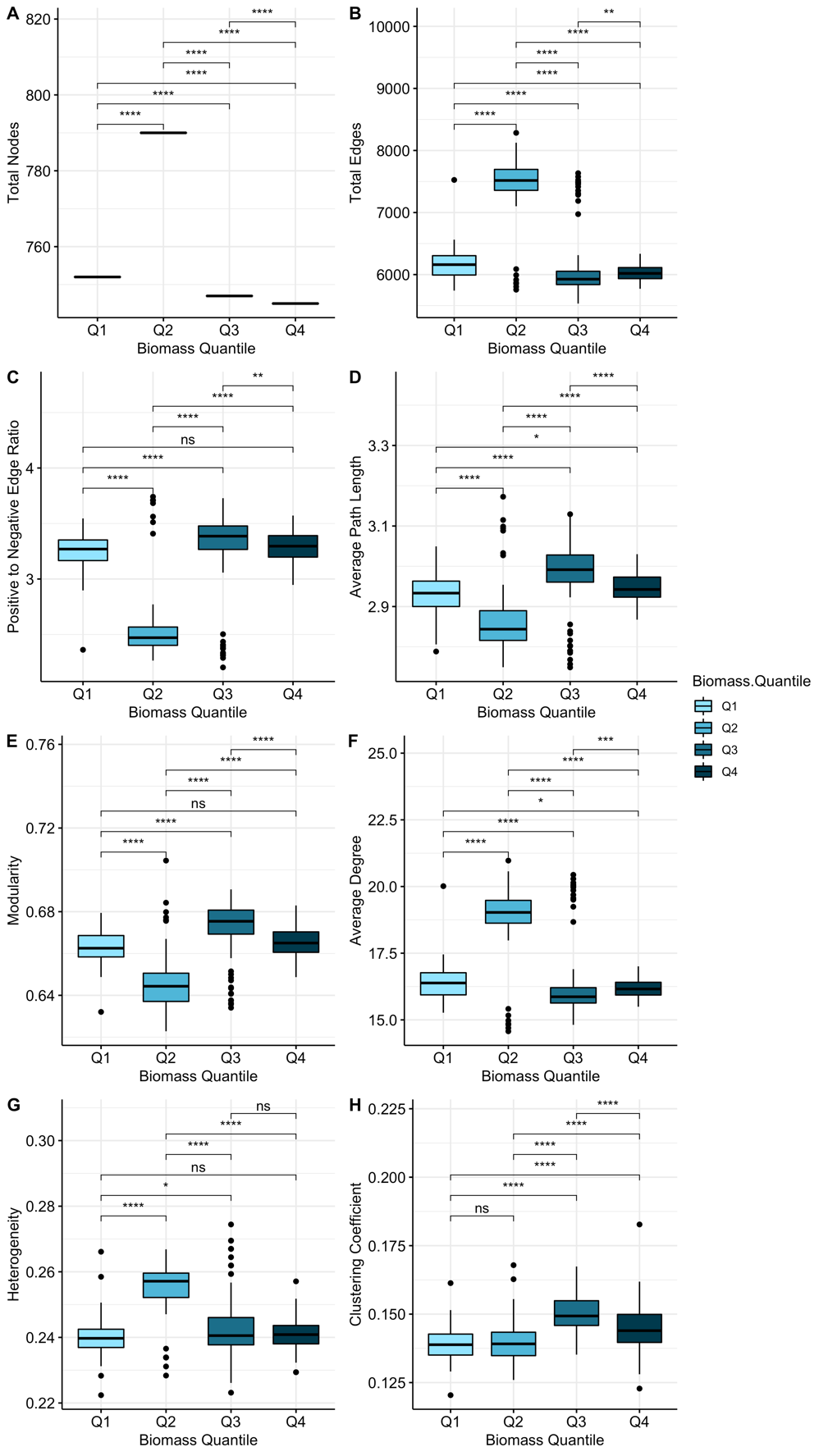
**

**S10 Fig. Box plots of network topology factors by biomass quantile at the species level.** Box plots of (A) total nodes, (B) total edges, (C) positive to negative edge ratio, (D) average path length, (E) modularity, (F) average degree, (G) heterogeneity, and (H) clustering coefficient for all biomass quantiles (Q1, Q2, Q3, Q4) with bacteria classified at the species level. Pairwise significance was tested with the Wilcoxon test: ns: not significant, *: p <= 0.05, **: p <= 0.01, ***: p<=0.001, ****: p <= 0.0001.
