# Supplementary material for: Investigating the relationship between microbial network features of giant kelp “seedbank” cultures and subsequent farm performance
Source: PLoS One. 2024 Mar 27;19(3):e0295740. doi: 10.1371/journal.pone.0295740 (PMC10971754; doi:10.1371/journal.pone.0295740)
Supplement: S1 Table — Network topology factors recorded for LC gametophytes (n = 308) with bacteria classified at four taxonomic levels: order, family, genus, and species. LC gametophytes were divided into four biomass quantiles and a summary of all data is presented here. For each taxonomic level, we randomly sampled 50 individuals from each quantile 100 times to create representative networks. Values have been rounded to four significant figures. (*) Used in regression model. (DOCX) [file pone.0295740.s011.docx]

| **Taxonomic Level** | **Network Topology Factor** | **Q1** | **Q2** | **Q3** | **Q4** |
| --- | --- | --- | --- | --- | --- |
| Order | Total Nodes | 60.23±12.32 | 55.06±11.44 | 48.31±10.59 | 52.37±11.20 |
| Order | Total Edges | 76.92±26.58 | 65.08±28.91 | 61.47±22.92 | 69.15±24.0 |
| Order | Positive Edges | 68.47±21.52 | 58.48±22.90 | 56.55±18.68 | 63.35±20.41 |
| Order | Negative Edges | 8.450±5.529 | 6.600±6.291 | 4.920±4.598 | 5.800±3.975 |
| Order | Positive / Negative Edge Ratio* | 13.38±11.52 | 17.12±12.08 | 14.25±11.42 | 17.99±11.92 |
| Order | Positive / Total Edge Ratio | 0.9055±0.05072 | 0.9188±0.05046 | 0.9381±0.05344 | 0.9269±0.03634 |
| Order | Negative / Total Edge Ratio | 0.09450±0.05072 | 0.08124±0.05046 | 0.06192±0.05344 | 0.07309±0.03634 |
| Order | Average Path Length* | 3.927±0.8294 | 3.498±1.236 | 3.401±0.8584 | 3.372±0.8062 |
| Order | Modularity* | 0.7755±0.03852 | 0.7723±0.04621 | 0.7672±0.03230 | 0.7398±0.03647 |
| Order | Average Degree | 2.468±0.4588 | 2.259±0.5351 | 2.456±0.4492 | 2.557±0.4296 |
| Order | Heterogeneity* | 0.3071±0.02037 | 0.3040±0.02499 | 0.3245±0.02405 | 0.3266±0.02673 |
| Order | Clustering Coefficient* | 0.1323±0.07696 | 0.1082±0.07814 | 0.1555±0.08380 | 0.1365±0.07386 |
| Family | Total Nodes | 151.8±6.495 | 150.2±7.113 | 138.6±4.467 | 143.0±8.932 |
| Family | Total Edges | 264.6±76.85 | 262.6±68.98 | 226.3±32.54 | 247.1±69.43 |
| Family | Positive Edges | 231.0±54.07 | 237.0±50.76 | 206.4±24.56 | 221.4±47.65 |
| Family | Negative Edges | 33.58±23.33 | 25.58±18.97 | 19.88±9.18 | 25.67±22.51 |
| Family | Positive / Negative Edge Ratio* | 8.682±2.946 | 12.39±5.869 | 11.37±2.941 | 12.29±5.436 |
| Family | Positive / Total Edge Ratio | 0.8844±0.04253 | 0.9115±0.03801 | 0.9143±0.02235 | 0.9083±0.04703 |
| Family | Negative / Total Edge Ratio | 0.1156±0.04253 | 0.08852±0.03801 | 0.08569±0.02235 | 0.09175±0.04703 |
| Family | Average Path Length | 5.375±1.038 | 5.180±0.9200 | 5.069±0.5308 | 4.990±0.7665 |
| Family | Modularity* | 0.8421±0.03459 | 0.8428±0.03151 | 0.8202±0.01752 | 0.8254±0.02740 |
| Family | Average Degree | 3.453±0.8370 | 3.466±0.7291 | 3.258±0.3687 | 3.415±0.7030 |
| Family | Heterogeneity* | 0.2462±0.02006 | 0.2464±0.01579 | 0.2488±0.01717 | 0.2563±0.01563 |
| Family | Clustering Coefficient* | 0.1800±0.02530 | 0.1781±0.02741 | 0.1613±0.02421 | 0.1651±0.02953 |
| Genus | Total Nodes | 476.0±0.000 | 484.0±0.000 | 461.0±0.000 | 471.0±0.000 |
| Genus | Total Edges | 2739±335.6 | 2855±238.9 | 2242±377.8 | 2324±417.6 |
| Genus | Positive Edges | 2151±201.4 | 2228±148.6 | 1813±229.4 | 1908±252.3 |
| Genus | Negative Edges | 588.5±137.5 | 626.9±95.46 | 428.9±152.4 | 416.5±170.4 |
| Genus | Positive / Negative Edge Ratio* | 3.864±0.9146 | 3.643±0.6006 | 4.534±0.9049 | 5.057±1.219 |
| Genus | Positive / Total Edge Ratio | 0.7887±0.0307 | 0.7820±0.0208 | 0.8139±0.0334 | 0.8273±0.0386 |
| Genus | Negative / Total Edge Ratio | 0.2113±0.03066 | 0.2180±0.02084 | 0.1861±0.03337 | 0.1727±0.03861 |
| Genus | Average Path Length | 3.093±0.1586 | 3.088±0.1125 | 3.289±0.1860 | 3.253±0.2052 |
| Genus | Modularity | 0.6893±0.02833 | 0.6817±0.01986 | 0.7260±0.03322 | 0.7173±0.03542 |
| Genus | Average Degree | 11.51±1.410 | 11.80±0.9872 | 9.727±1.639 | 9.869±1.773 |
| Genus | Heterogeneity* | 0.2338±0.01620 | 0.2365±0.01200 | 0.2226±0.01733 | 0.2199±0.01909 |
| Genus | Clustering Coefficient* | 0.1472±0.01207 | 0.1478±0.01174 | 0.1623±0.02524 | 0.1547±0.02265 |
| Species | Total Nodes | 752.0±0.000 | 790.0±0.000 | 747.0±0.000 | 745.0±00.00 |
| Species | Total Edges | 6160±245.1 | 7448±483.9 | 6102±526.3 | 6027±141.1 |
| Species | Positive Edges | 4709±159.8 | 5327±241.6 | 4648±240.1 | 4620±108.8 |
| Species | Negative Edges | 1451±109.3 | 2121±258.0 | 1455±297.4 | 1407±60.32 |
| Species | Positive / Negative Edge Ratio | 3.256±0.1609 | 2.548±0.3143 | 3.269±0.3725 | 3.289±0.1352 |
| Species | Positive / Total Edge Ratio | 0.7646±0.009726 | 0.7163±0.02040 | 0.7636±0.02470 | 0.7666±0.007416 |
| Species | Negative / Total Edge Ratio | 0.2354±0.009726 | 0.2837±0.02040 | 0.2364±0.02470 | 0.2334±0.007416 |
| Species | Average Path Length | 2.933±0.04780 | 2.861±0.07925 | 2.980±0.08240 | 2.948±0.03552 |
| Species | Modularity | 0.6631±0.007506 | 0.6455±0.01266 | 0.6721±0.01332 | 0.6652±0.007132 |
| Species | Average Degree | 16.38±0.6519 | 18.86±1.225 | 16.34±1.409 | 16.18±0.3787 |
| Species | Heterogeneity | 0.2400±0.005982 | 0.2554±0.007691 | 0.2430±0.009911 | 0.2405±0.004910 |
| Species | Clustering Coefficient* | 0.1390±0.005656 | 0.1398±0.007559 | 0.1501±0.005903 | 0.1447±0.008012 |

**S1 Table. Summary of network topology factors.** Network topology factors recorded for LC gametophytes (n = 308) with bacteria classified at four taxonomic levels: order, family, genus, and species. LC gametophytes were divided into four biomass quantiles and a summary of all data is presented here. For each taxonomic level, we randomly sampled 50 individuals from each quantile 100 times to create representative networks. Values have been rounded to four significant figures. (*) Used in regression model.
