# Supplementary material for: Investigating the relationship between microbial network features of giant kelp “seedbank” cultures and subsequent farm performance
Source: PLoS One. 2024 Mar 27;19(3):e0295740. doi: 10.1371/journal.pone.0295740 (PMC10971754; doi:10.1371/journal.pone.0295740)
Supplement: S2 Table — P-value and odds ratio values for network topology factors used in proportional odds logistic regression (POLR) model. Models were as follows: (Order) Biomass Quantile ~ Positive to Negative Edge Ratio + Average Path Length + Modularity + Heterogeneity + Clustering Coefficient, (Family) Biomass Quantile ~ Positive to Negative Edge Ratio + Modularity + Heterogeneity + Clustering Coefficient, (Genus) Biomass Quantile ~ Positive to Negative Edge Ratio + Heterogeneity + Clustering Coefficient, and (Species) Biomass Quantile ~ Clustering Coefficient. (DOCX) [file pone.0295740.s012.docx]

| **Taxonomic Level** | **Network Topology Factor** | **P-Value** | **Odds Ratio** |
| --- | --- | --- | --- |
| Order | Clustering Coefficient | 2.64e-4 | 3.52e+3 |
| Order | Positive to Negative Edge Ratio | 3.10e-3 | 1.04 |
| Order | Heterogeneity | 2.54e-5 | 1.00e+9 |
| Order | Modularity | 7.40e-9 | 5.07e-8 |
| Order | Average Path Length | 2.16e-4 | 5.73e-1 |
| Family | Clustering Coefficient | 3.13e-3 | 2.33e-6 |
| Family | Positive to Negative Edge Ratio | 4.67e-10 | 1.22 |
| Family | Heterogeneity | 5.69e-3 | 6.34e+9 |
| Family | Modularity | 3.92e-5 | 2.22e-10 |
| Genus | Clustering Coefficient | 8.08e-3 | 2.46e-8 |
| Genus | Positive to Negative Edge Ratio | 3.30e-10 | 4.65 |
| Species | Clustering Coefficient | 1.84e-10 | 1.60e+37 |

**S2 Table. Summary of p-value and odds ratio values.** P-value and odds ratio values for network topology factors used in proportional odds logistic regression (POLR) model. Models were as follows: (Order) Biomass Quantile ~ Positive to Negative Edge Ratio + Average Path Length + Modularity + Heterogeneity + Clustering Coefficient, (Family) Biomass Quantile ~ Positive to Negative Edge Ratio + Modularity + Heterogeneity + Clustering Coefficient, (Genus) Biomass Quantile ~ Positive to Negative Edge Ratio + Heterogeneity + Clustering Coefficient, and (Species) Biomass Quantile ~ Clustering Coefficient.
