# Supplementary material for: Investigating the relationship between microbial network features of giant kelp “seedbank” cultures and subsequent farm performance
Source: PLoS One. 2024 Mar 27;19(3):e0295740. doi: 10.1371/journal.pone.0295740 (PMC10971754; doi:10.1371/journal.pone.0295740)
Supplement: S3 Table — Hub taxa with a Kleinberg’s centrality score of over 0.5. M. pyrifera gametophytes from all four populations (AQ, CI, CP, and LC). Representative networks were generated for the microbial communities of each population. Taxa were then given a score to quantify their role as a hub taxa. Taxa from the order and family levels that scored over 0.5 are recorded here. *,+ denotes hub taxa found in more than one population with a score over 0.5. (DOCX) [file pone.0295740.s013.docx]

| **Taxonomic Level** | **Taxa** | **Hub Score** | **Population** |
| --- | --- | --- | --- |
| Order | NIASMIV (Gammaproteobacteria) | 1 | AQ |
| Order | Chthoniobacterales^+^ | 0.94 | AQ |
| Order | Chlorobiales | 1 | CI |
| Order | Ignavibacteriales | 0.56 | CI |
| Order | Bifidobacteriales | 1 | CP |
| Order | Myxococcales | 0.95 | CP |
| Order | Chthoniobacterales^+^ | 0.83 | CP |
| Order | Frankiales | 1 | LC |
| Order | Subsection III (Cyanobacteria) | 0.95 | LC |
| Order | Kineosporiales | 0.87 | LC |
| Order | Subsection IV (Cyanobacteria) | 0.79 | LC |
| Order | Micromonosporales | 0.62 | LC |
| Order | Solirubrobacterales | 0.56 | LC |
| Family | Iamiaceae | 1 | AQ |
| Family | Cryptosporangiaceae* | 0.91 | AQ |
| Family | Micrococcaceae | 0.77 | AQ |
| Family | Chlorobiaceae | 1 | CI |
| Family | Ignavibacteriales Incertae Sedis | 0.99 | CI |
| Family | Sphingomonadaceae | 0.54 | CI |
| Family | Ectothiorhodospiraceae | 1 | CP |
| Family | Granulosicoccaceae | 0.92 | CP |
| Family | Family I (Cyanobacteria, Subsection III) | 1 | LC |
| Family | Kineosporiaceae | 0.97 | LC |
| Family | Cryptosporangiaceae* | 0.96 | LC |
| Family | Family I (Cyanobacteria, Subsection IV) | 0.92 | LC |
| Family | Micromonosporaceae | 0.72 | LC |
| Family | Moritellaceae | 0.70 | LC |
| Family | Bdellovibrionaceae | 0.69 | LC |
| Family | Caedibacter caryophilus group (Rickettsiales) | 0.63 | LC |
| Family | Mycobacteriaceae | 0.62 | LC |
| Family | Staphylococcaceae | 0.61 | LC |

**S3 Table. Hub taxa by population.** Hub taxa with a Kleinberg’s centrality score of over 0.5. *M. pyrifera* gametophytes from all four populations (AQ, CI, CP, and LC). Representative networks were generated for the microbial communities of each population. Taxa were then given a score to quantify their role as a hub taxa. Taxa from the order and family levels that scored over 0.5 are recorded here. *,+ denotes hub taxa found in more than one population with a score over 0.5.
