# Supplementary material for: Investigating the relationship between microbial network features of giant kelp “seedbank” cultures and subsequent farm performance
Source: PLoS One. 2024 Mar 27;19(3):e0295740. doi: 10.1371/journal.pone.0295740 (PMC10971754; doi:10.1371/journal.pone.0295740)
Supplement: S4 Table — Resulting p-values for Kruskal-Wallis rank sum test comparing network features across all biomass quantiles. Results recorded for networks built with bacteria classified at the order, family, genus, and species levels. (DOCX) [file pone.0295740.s014.docx]

| **Network Topology Factor** | **P-Value** | | | |
| --- | --- | --- | --- | --- |
|  | Order | Family | Genus | Species |
| Total Nodes | 3.743e-15 | 2.2e-16 | 2.2e-16 | 2.2e-16 |
| Total Edges | 6.259e-07 | 7.089e-09 | 2.2e-16 | 2.2e-16 |
| Positive to Negative Edge Ratio | 3.603e-05 | 9.052e-11 | 2.051e-15 | 2.2e-16 |
| Average Path Length | 1.528e-05 | 0.0001952 | 1.069e-10 | 2.2e-16 |
| Modularity | 1.049e-10 | 8.513e-14 | 2.2e-16 | 2.2e-16 |
| Average Degree | 0.000253 | 0.002852 | 1.916e-14 | 2.2e-16 |
| Heterogeneity | 4.2e-13 | 4.324e-05 | 2.947e-10 | 2.2e-16 |
| Clustering Coefficient | 0.0002311 | 5.485e-07 | 7.147e-07 | 2.2e-16 |

**S4 Table.** **Significant differences between network features across biomass outcomes.** Resulting p-values for Kruskal-Wallis rank sum test comparing network features across all biomass quantiles. Results recorded for networks built with bacteria classified at the order, family, genus, and species levels.
