# Supplementary material for: Investigating the relationship between microbial network features of giant kelp “seedbank” cultures and subsequent farm performance
Source: PLoS One. 2024 Mar 27;19(3):e0295740. doi: 10.1371/journal.pone.0295740 (PMC10971754; doi:10.1371/journal.pone.0295740)
Supplement: S5 Table — Number of samples and number of taxa that were used in network construction for all four biomass quantiles from the LC population. (DOCX) [file pone.0295740.s015.docx]

|  | **Biomass Quantile** | | | | | | | |
| --- | --- | --- | --- | --- | --- | --- | --- | --- |
| **Taxonomic Level** | Q1 | | Q2 | | Q3 | | Q4 | |
|  | Sample Size | Number of Taxa | Sample Size | Number of Taxa | Sample Size | Number of Taxa | Sample Size | Number of Taxa |
| Order | 77 | 82 | 78 | 81 | 76 | 74 | 77 | 78 |
| Family | 77 | 163 | 78 | 163 | 76 | 156 | 77 | 162 |
| Genus | 77 | 476 | 78 | 484 | 76 | 461 | 77 | 471 |
| Species | 77 | 752 | 78 | 790 | 76 | 747 | 77 | 745 |

**S5 Table.** **Sample size and number of taxa.** Number of samples and number of taxa that were used in network construction for all four biomass quantiles from the LC population.
